# Chronic Heart Failure: Diagnosis and Management beyond LVEF Classification

**DOI:** 10.3390/jcm11061718

**Published:** 2022-03-19

**Authors:** Filippos Triposkiadis, Randall C. Starling

**Affiliations:** 1 Department of Cardiology, University of Thessaly, Larissa University General Hospital, 41110 Larissa, Greece; 2 Cleveland Clinic Lerner College of Medicine, Case Western Reserve University, Cleveland, OH 44195, USA

The classification, phenotyping, and management of heart failure (HF) has been based on the left ventricular (LV) ejection fraction (LVEF) [1]. In the recent Universal Definition, HF was designated as HF with reduced LVEF (HFrEF, LVEF ≤ 40%); HF with mildly reduced LVEF (HFmrEF, 41% to 49%); HF with preserved LVEF (HFpEF ≥ 50%); and HF with improved LVEF (HFimpEF, LVEF ≥ 40% at baseline associated with a ≥10-point increase from baseline and a repeat measurement of >40%) [2]. Others proposed a modified, LVEF-based HF classification using sex-specific cut-offs to define as “normal” an LVEF ≥ 55% in men and ≥60% in women [3].

HF is a very complex syndrome, and it is unrealistic to predicate its classification on a single biomarker [4]. Moreover, a mandatory prerequisite for the use of LVEF ranges to delineate HF phenotypes is an undisputed agreement on the LVEF normal range which has not been reached. According to the American Society of Echocardiography and the European Association of Cardiovascular Imaging, the normal LVEF range is 52–72% in men and 54–74% in women [5]. However, recent evidence contradicts this delineation. A study in which physician-reported LVEF (403,977 echocardiograms from 203,135 patients) were linked to all-cause mortality from the US healthcare system, and was independently validated in a dataset (45,531 echocardiograms and 35,976 patients) from New Zealand, demonstrated that the overall, unadjusted hazard ratios for mortality showed a U-shaped relationship for LVEF with a nadir of risk at an LVEF of 60–65%, and similar results were obtained after further adjustments for age, sex, or HF [6]. Slightly different but towards the same direction were the findings of another study including approximately 500,000 participants, which reported that in both women and men, mortality was lowest at an LVEF level of 65.0–69.9% [7]. In the same study in females, an increased risk for cardiovascular-related mortality persisted to an LVEF level of 60.0–64.9%, whereas in males the corresponding LVEF level was lower (55.0–59.9%) [7].

Moreover, the controversies regarding the delineation of normal range show there is substantial discordance between the various imaging modalities in LVEF measurement among expert laboratories [8]. Importantly, even if echocardiography, the most widely available cardiovascular imaging, is used for LVEF determination, significant intraobserver and interobserver variability usually occurs [9].

Equally disturbing with the variability in measurements is the vague physiological significance of LVEF. The LVEF, which equals the LV stroke volume (LVSV) divided by the LV end-diastolic volume (LVEDV) and is usually expressed as a percentage, is inappropriately assumed to be an index of myocardial contractility [10]. However, it has been largely disregarded that (a) LVEF is influenced by the loading conditions (preload and afterload) and cannot represent myocardial contractility without knowledge of LV loads; and (b) structural changes leading to increases or decreases in LVEDV will have a major impact on the LVEF at any given level of contractility and SV [11]. Thus, LVEF has severe limitations when used as a measure of LV systolic function.

Finally, LVEF has long been used for the guidance of medical treatment in HF. In this regard, the lack of effective medications for HFpEF has represented for years a large and growing unmet need in cardiology [12]. However, compounds such as spironolactone, sacubitril/valsartan, and sodium–glucose cotransporter 2 inhibitors (SGLT-2i) have proved to be effective over a wide range of LVEF [13]. Moreover, recent evidence suggests that acute HF and advanced HF may occur at any level of LVEF, and prognosis is unrelated to LVEF [14].

To have a meaningful impact, classification systems should reflect current approaches in HF evaluation and management. Although the LVEF has served for a long time as the holy grail of HF, the time to be ejected as the primary classification system for this global pandemic has arrived [15,16].

## References

[1] McDonagh T.A., Metra M., Adamo M., Gardner R.S., Baumbach A., Böhm M., Burri H., Butler J., Čelutkienė J., Kathrine Skibelund A. (2021). 2021 ESC Guidelines for the diagnosis and treatment of acute and chronic heart failure. Eur. Heart J..

[2] Bozkurt B., Coats A.J., Tsutsui H., Abdelhamid C.M., Adamopoulos S., Albert N., Anker S.D., Atherton J., Böhm M., Zieroth S. (2021). Universal Definition and Classification of Heart Failure: A Report of the Heart Failure Society of America, Heart Failure Association of the European Society of Cardiology, Japanese Heart Failure Society and Writing Committee of the Universal Definition of Heart Failure. Eur. J. Heart Fail..

[3] Lam C.S.P., Solomon S.D. (2021). Classification of Heart Failure According to Ejection Fraction: JACC Review Topic of the Week. J. Am. Coll. Cardiol..

[4] Triposkiadis F., Butler J., Abboud F.M., Armstrong P.W., Adamopoulos S., Atherton J.J., Backs J., Bauersachs J., Burkhoff D., De Keulenaer G.W. (2019). The continuous heart failure spectrum: Moving beyond an ejection fraction classification. Eur. Heart J..

[5] Lang R.M., Badano L.P., Mor-Avi V., Afilalo J., Armstrong A., Ernande L., Flachskampf F.A., Foster E., Goldstein S.A., Kuznetsova T. (2015). Recommendations for cardiac chamber quantification by echocardiography in adults: An update from the American Society of Echocardiography and the European Association of Cardiovascular Imaging. Eur. Heart J.-Cardiovasc. Imaging.

[6] Wehner G.J., Jing L., Haggerty C.M., Suever J.D., Leader J.B., Hartzel D.N., Fornwalt B.K. (2020). Routinely reported ejection fraction and mortality in clinical practice: Where does the nadir of risk lie?. Eur. Heart J..

[7] Stewart S., Playford D., Scalia G.M., Currie P., Celermajer D.S., Prior D., Codde J., Strange G. (2021). Ejection fraction and mortality: A nationwide register-based cohort study of 499 153 women and men. Eur. J. Heart Fail..

[8] Pellikka P.A., She L., Holly T.A., Lin G., Varadarajan P., Pai R.G., Bonow R.O., Pohost G.M., Panza J.A., Berman D.S. (2018). Variability in Ejection Fraction Measured By Echocardiography, Gated Single-Photon Emission Computed Tomography, and Cardiac Magnetic Resonance in Patients with Coronary Artery Disease and Left Ventricular Dysfunction. JAMA Netw. Open.

[9] Butler J., Anker S.D., Packer M. (2019). Redefining Heart Failure with a Reduced Ejection Fraction. JAMA.

[10] Katz A.M., Rolett E.L. (2016). Heart failure: When form fails to follow function. Eur. Heart J..

[11] Konstam M.A., Abboud F.M. (2017). Ejection Fraction: Misunderstood and Overrated (Changing the Paradigm in Categorizing Heart Failure). Circulation.

[12] Roh J., Houstis N., Rosenzweig A. (2017). Why Don’t We Have Proven Treatments for HFpEF?. Circ. Res..

[13] Triposkiadis F., Xanthopoulos A., Starling R.C. (2021). Medical Treatment of Heart Failure: Ignore the Ejection Fraction and Treat All?. J. Card. Fail..

[14] Dunlay S., Roger V., Killian J., Weston S., Schulte P., Subramaniam A., Blecker S., Redfield M. (2021). Advanced Heart Failure Epidemiology and Outcomes: A Population-Based Study. JACC Heart Fail..

[15] Greenberg B., O’Connor C.M., Felker G.M. (2021). Classifying Heart Failure in the 21st Century: Matching Taxonomy with Science. JACC Heart Fail..

[16] Severino P., D’Amato A., Prosperi S., Dei Cas A., Mattioli A.V., Cevese A., Novo G., Prat M., Pedrinelli R., Raddino R. (2022). Do the Current Guidelines for Heart Failure Diagnosis and Treatment Fit with Clinical Complexity?. J. Clin. Med..

